# *Lactobacillus helveticus* SBT2171 Attenuates Experimental Autoimmune Encephalomyelitis in Mice

**DOI:** 10.3389/fmicb.2017.02596

**Published:** 2018-01-22

**Authors:** Maya Yamashita, Ken Ukibe, Yumi Matsubara, Tomohiro Hosoya, Fumihiko Sakai, Shigeyuki Kon, Yasunobu Arima, Masaaki Murakami, Hisako Nakagawa, Tadaaki Miyazaki

**Affiliations:** ^1^Milk Science Research Institute, Megmilk Snow Brand Co., Ltd., Saitama, Japan; ^2^Department of Probiotics Immunology, Institute for Genetic Medicine, Hokkaido University, Sapporo, Japan; ^3^Department of Pharmacy and Pharmaceutical Sciences, Fukuyama University, Fukuyama, Japan; ^4^Department of Molecular Neuroimmunology, Institute for Genetic Medicine, Hokkaido University, Sapporo, Japan

**Keywords:** *Lactobacillus helveticus* SBT2171, experimental autoimmune encephalomyelitis, multiple sclerosis, Th17 cells, interleukin-6

## Abstract

We recently reported that *Lactobacillus helveticus* SBT2171 (LH2171) inhibited the proliferation and inflammatory cytokine production of primary immune cells *in vitro*, and alleviated collagen-induced arthritis (CIA) in mice, a model of human rheumatoid arthritis (RA). In this study, we newly investigated whether LH2171 could relieve the severity of experimental autoimmune encephalomyelitis (EAE), a murine model of multiple sclerosis (MS), which is an autoimmune disease, but develop the symptoms by different mechanisms from RA. In MS and EAE, main cause of the disease is the abnormality in CD4^+^ T cell immunity, whereas in RA and CIA, is that in antibody-mediated immunity. The intraperitoneal administration of LH2171 significantly decreased the incidence and clinical score of EAE in mice. LH2171 also reduced the numbers of pathogenic immune cells, especially Th17 cells, in the spinal cord at the peak stage of disease severity. Interestingly, before the onset of EAE, LH2171 administration remarkably decreased the ratio of Th17 cells to CD4^+^ T cells in the inguinal lymph nodes (LNs), where pathogenic immune cells are activated to infiltrate the central nervous system, including the spinal cord. Furthermore, the expression of interleukin (IL)-6, an inflammatory cytokine essential for Th17 differentiation, decreased in the LNs of LH2171-administered mice. Moreover, LH2171 significantly inhibited IL-6 production *in vitro* from both DC2.4 and RAW264.7 cells, model cell lines of antigen-presenting cells. These findings suggest that LH2171 might down-regulate IL-6 production and the subsequent Th17 differentiation and spinal cord infiltration, consequently alleviating EAE symptoms.

## Introduction

Multiple sclerosis is an autoimmune disease of the CNS characterized by demyelination, inflammatory lesions, axonal damage, activation of IFN-γ-producing Th1 cells and IL-17-producing Th17 cells, inappropriate activation of innate immune cells (macrophages, dendritic cells, neutrophils, and microglia), and aberrant production of cytokines and chemokines (Ryu et al., 2013; Cheng and Chen, 2014; Serada et al., 2016). Patients with MS experience abnormalities with respect to sensation, mobility, balance, sphincter function, vision, and cognition (Brownlee et al., 2017). The incidence of MS has been on the rise, with a global prevalence estimated at 2.3 million in 2013, representing an increase of 0.2 million people from 5 years earlier (Ontaneda et al., 2017), and is a leading cause of neurological disabilities in young adults (Compston and Coles, 2002). Th1 and Th17 cells are implicated in the development of EAE, an animal model of MS (Goverman, 2009). The pathogenesis of both MS and EAE is associated with the overexpression of cytokines, including IL-12, IFN-γ, IL-6, IL-1β, IL-21, and IL-23, which function in part to promote the differentiation of effector Th1 and Th17 cells (Bhat and Steinman, 2009; Goverman, 2009; Domingues et al., 2010; Segal, 2010).

It has recently been reported that LAB have the beneficial effects on human health not only to improve the environment of the intestine, but also to have an influence on immune functions. For instance, the intake of *Lactobacillus gasseri* SBT2055 increased hemagglutination- inhibition titers against influenza viruses (IFVs) after vaccination as compared to the intake of placebo by healthy volunteers (Nishihira et al., 2016). SBT2055 was found to effectively protect against influenza A virus infection by suppressing viral replication through induction of the expression of antiviral genes in mice (Nakayama et al., 2014; Miyazaki, 2015). Oral administration of *Lactobacillus brevis* KB290 to mice also alleviates clinical symptoms following influenza virus infection by the enhancement of IFN-α production and augmentation of IFV-specific IgA production (Waki et al., 2014). Moreover, the beneficial effects of LAB on allergy symptoms have recently been reported. The intake of *Lactobacillus acidophilus* L-92 reduced subjective symptoms in adult patients with atopic dermatitis (Yamamoto et al., 2016). *Lactobacillus casei* strain Shirota suppresses systemic anaphylaxis in a food allergy model mice (Shida et al., 2002), and modifies allergen-induced immune responses in allergic rhinitis in human (Ivory et al., 2008). In addition, some reports have demonstrated the effect of LAB on autoimmune diseases (Kato et al., 1998; So et al., 2008; Lavasani et al., 2010; Amdekar et al., 2011; Berer et al., 2011) including MS and EAE, although the specific mechanisms by which LAB exert these alleviative effects remain to be elucidated.

Here, we focused on the immune-regulatory activity of the LAB, LH2171. LH2171, a strain of lactobacilli that originates from dairy products, exhibits high protease activity, and is a common starter bacterial strain in the production of a Gouda-type cheese (Sasaki et al., 1996). In our previous study, LH2171 strongly inhibited the proliferation of primary murine immune cells among 41 species of LAB strains, and suppressed the production of LPS-stimulated inflammatory cytokines (IL-6 and IL-1β) from the immune cells *in vitro* (Yamashita et al., 2014). Furthermore, *in vivo* administration of LH2171 suppressed the incidence and development of rheumatoid arthritis, one of the major autoimmune diseases, in a murine model (Hosoya et al., 2014; Yamashita et al., 2017).

In the present study, to discover a further beneficial property of LH2171 for an excessive immune function, we investigated its alleviating effects in an EAE model. These findings could help to elucidate the mechanisms by which LAB regulate immune function and should contribute to the promotion and development of LAB-based prevention or treatment strategies for immunological diseases.

## Materials and Methods

### Bacterial Strain

*Lactobacillus helveticus* SBT2171 (LH2171) was isolated by Megmilk Snow Brand (Tokyo, Japan). LH2171 was inoculated into Lactobacilli MRS broth (BD Biosciences, San Jose, CA, United States) and cultivated for 16 h at 37°C. Following incubation, the cells were harvested by centrifugation at 8,000 × *g* for 10 min. The cells were washed twice with saline and once with distilled water and freeze-dried. Freeze-dried bacterial cells were resuspended in phosphate buffered saline (PBS) at 10 mg/mL and heat-killed at 80°C for 30 min.

### Induction of EAE and Administration of LH2171

Female SJL/J mice (5 weeks old) were used to assess the immunosuppressive effect of LH2171 on EAE. The mice (Charles River Japan, Yokohama, Japan) received sterile water and standard chow (Labo MR Stock; Nosan Corporation, Yokohama, Japan) *ad libitum*. All experiments involving mice were carried out in accordance with the guidelines of the Bioscience Committee of Hokkaido University and were approved by the Animal Care and Use Committee of Hokkaido University.

To induce EAE (Greer et al., 1996; Stromnes and Goverman, 2006; Arima et al., 2012), the mice were immunized by intradermal injection of 100 μg of PLP139–151 (HSLGKWLGHPDKF), which was dissolved in 100 μL of PBS and emulsified in 100 μL of CFA (Difco Laboratories, Detroit, MI, United States) containing 100 μg of *Mycobacterium tuberculosis* H37Ra (an avirulent strain) at the base of the tail, in addition to two intraperitoneal injections of 400 ng of pertussis toxin (List Laboratories, Campbell, CA, United States) on days 0 and 2. EAE mice were divided into two groups: (1) mice receiving LH2171 (LH2171 group) and (2) mice receiving vehicle only (control group). In the LH2171 group, the mice received 1 mg of LH2171 by intraperitoneal injection three times a week from 3 weeks before immunization and then daily after the immunization. Using the same treatment regimen, mice in the control group received the same volume of PBS only by intraperitoneal injection.

The incidence and disease severity of EAE were assessed after the immunization. Disease severity in each mouse was scored on a scale of 0–7: (0) no signs of clinical disease; (1) paralyzed tail; (2) gait disturbance; (3) mild paresis of the hind limbs; (4) paralysis of the hind limbs; (5) paralysis of the extremities, but movement still possible; (6) moribund state; and (7) dead.

### Histopathological Assessment

The spinal cord was obtained on day 42 from the mice with EAE after cardiac perfusion with PBS. The tissues were fixed in 10% paraformaldehyde, decalcified in ethylenediaminetetraacetic acid (Sigma–Aldrich, Tokyo, Japan), and embedded in paraffin. The samples were prepared and stained with haematoxylin and eosin (Merck Millipore, Guyancourt, France).

### Gene Expression Analysis

Total RNA was isolated from the inguinal LNs of EAE mice using the TRIzol reagent (Life Technologies, Thermo Scientific, Waltham, MA, United States). RNA was reverse-transcribed into complementary DNA using the ReverTra Ace qPCR kit (TOYOBO, Osaka, Japan). Real-time PCR was performed using the KAPA SYBR FAST Universal qPCR Kit (Kapa Biosystems, Boston, MA, United States) according to the manufacturer’s instructions. Sequences of the PCR primers are shown in **Table 1**. Data were normalized to *Actb* gene expression.

**Table 1:** Primers used in the gene expression analysis.

Gene symbol	Forward primer 5′→3′	Reverse primer 5′→3′
*Actb*	GGCTGTATTCCCCTCCATCG	CCAGTTGGTAACAATGCCATGT
*Ifng*	CTGCAGAGCCAGATTATCTC	CCTGTGGGTTGTTGACCTCA
*Il17a*	GCTCCAGAAGGCCCTCAGA	CTTTCCCTCCGCATTGACA
*Il6*	CGTGGAAATGAGAAAAGAGTTGTGC	TGGTACTCCAGAAGACCAGAGGA
*Tgfb1*	CGGCAGTGGCTGAACCAAGGA	GACGTTTGGGGCTGATCCCGTT
*Il23p19*	GAGCAACTTCACACCTCCCT	TAGAACTCAGGCTGGGCAT
*Il1b*	CCCTGCAGCTGGAGAGTGTGGA	TGTGCTCTGCTTGTGAGGTGCTG
*Il10*	GCCCCAGGCAGAGAAGCATGG	GGGGAGAAATCGATGACAGCGCC
*Foxp3*	TTCATGCATCAGCTCTCCAC	CTGGACACCCATTCCAGACT

### Cell Staining and Flow Cytometric Analysis

To prepare single-cell suspensions from the inguinal LNs, the LNs of mice were collected and mechanically disrupted in RPMI 1640 culture medium (Wako, Osaka, Japan) containing 10% heat-inactivated fetal bovine serum (Gibco, Thermo Fisher Scientific, Waltham, MA, United States), 100 U/mL of penicillin, 100 μg/mL of streptomycin (Sigma–Aldrich, St. Louis, MO, United States), and 0.05 mM of 2-mercaptoethanol. Cell suspensions were filtered through a 100-μm cell strainer (BD Biosciences), washed twice with the medium, and resuspended in the cell culture medium. To prepare single-cell suspensions from the spinal cord, the spinal cord was collected after cardiac perfusion with PBS and digested with collagenase D (2.5 mg/mL; Roche Diagnostics, Mannheim, Germany) and DNase I (50 μg/mL; Roche Diagnostics) at 37°C for 45 min. To remove myelin debris from the single-cell suspensions, the tissue was passed through 100-μm cell strainers (BD Biosciences, San Jose, CA, United States), followed by magnetic separation with Myelin Removal Beads II (Miltenyi-Biotec, Bergisch Gladbach, Germany) according to the manufacturer’s instructions. The following markers were used to determine the type of immune cells: CD4^+^ T cells (CD3^+^ CD4^+^), Th1 cells (CD3^+^ CD4^+^ IFN-γ^+^), Th17 cells (CD3^+^ CD4^+^ IL-17A^+^). The following fluorochrome-labeled anti-mouse antibodies were used for fluorescence-activated cell sorting analyses: anti-CD3 (145-2C11), anti-CD4 (GK1.5), anti-IL-17A (TC11-18H10.1), and anti-IFN-γ (XMG1.2) (BioLegend, San Diego, CA, United States). For Th1 and Th17 staining, the cells were stimulated with phorbol 12-myristate 13-acetate (50 ng/mL) and ionomycin (500 ng/mL) in the presence of brefeldin A (1 μL/mL) (GolgiPlug; BD Biosciences, Heidelberg, Germany) for 5.5 h. Subsequently, the cells were stained intracellularly with anti-IFN-γ and anti-IL-17A antibodies using the BD Cytofix/Cytoperm reagents (BD Biosciences) according to the manufacturer’s protocols. Cytometric data were acquired using a FACSCanto II flow cytometer and analyzed with FACSDiva software (BD Biosciences).

### Cell Lines and Culture Conditions

DC2.4 cells, an immature DC cell line, were cultured in RPMI 1640 culture medium (Wako) supplemented with 10% heat-inactivated fetal bovine serum (Gibco), 0.05 mM 2-mercaptoethanol, 10 mM HEPES, 1 mM sodium pyruvate, 100 μM minimal essential medium (MEM) non-essential amino acids, 100 U/mL of penicillin, and 100 μg/mL of streptomycin (Wako). RAW 264.7 cells, a mouse macrophage cell line, were cultured in Dulbecco’s modified Eagle medium supplemented with 10% heat-inactivated fetal bovine serum, 100 μM MEM non-essential amino acids (Sigma–Aldrich), 100 U/mL of penicillin, and 100 μg/mL of streptomycin (Sigma–Aldrich). DC2.4 cells (5 × 10^4^ cells) and RAW 264.7 cells (5 × 10^4^ cells) were seeded in 96-well culture plates, respectively, and incubated to adhere overnight at 37°C in a humidified atmosphere of 5% CO_2_. After the incubation, the cells were treated with 10 μg/mL LH2171 and incubated for a further 4 h. Then, DC2.4 cells were stimulated with LPS from *Escherichia coli* 055:H5 (Sigma–Aldrich) at a final concentration of 1 μg/mL, and RAW 264.7 cells were stimulated with LPS from *E. coli* 0111:B4 (Sigma–Aldrich) at a final concentration of 10 μg/mL for 20 h. In all experiments, the cells were grown to 80–90% confluence.

### Cytokine Assay

The media of cultured DC2.4 cells and RAW 264.7 cells were harvested and stored at -80°C. The secretion of IL-6 in the culture cell media was measured with enzyme-linked immunosorbent assay kits (Mouse IL-6 ELISA MAX^TM^ Standard, BioLegend). The cytokine concentration was evaluated according to the manufacturer’s protocol.

### Statistical Analysis

The difference in EAE incidence between groups was assessed with the log-rank test. The Tukey–Kramer test was used to assess differences in IL-6 production from LPS-stimulated DC2.4 and RAW264.7 cells, and Student’s *t*-test was used for evaluations of other measured values. The log-rank test was performed using EZR (Saitama Medical Center, Jichi Medical University, Saitama, Japan), which is a graphical user interface for R (version 1.21; the R Foundation for Statistical Computing, Vienna, Austria). Other analyses were performed using StatView version 5.0 (SAS Institute, Cary, NC, United States). *P* values of < 0.05 were considered statistically significant. Statistical differences between the groups analyzed using the Tukey–Kramer test (*P* < 0.05) are represented by different letters (a–c).

## Results

### LH2171 Decreased the Incidence and Alleviated the Symptoms of EAE in Mice

**FIGURE 1:**
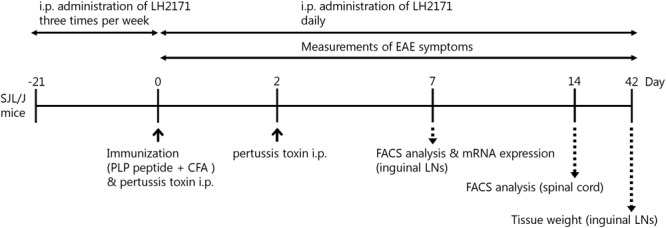
Experimental schedule. Female SJL/J mice (5 weeks old) were immunized by intradermal injection with 100 μg of PLP139–151 (HSLGKWLGHPDKF) emulsified with CFA, in addition to two intraperitoneal injections of 400 ng of pertussis toxin on days 0 and 2. For assessment of LH2171, the mice were injected with 100 μL of PBS containing 1 mg of LH2171 intraperitoneally three times a week from 3 weeks before immunization and then daily after the immunization.

**FIGURE 2:**
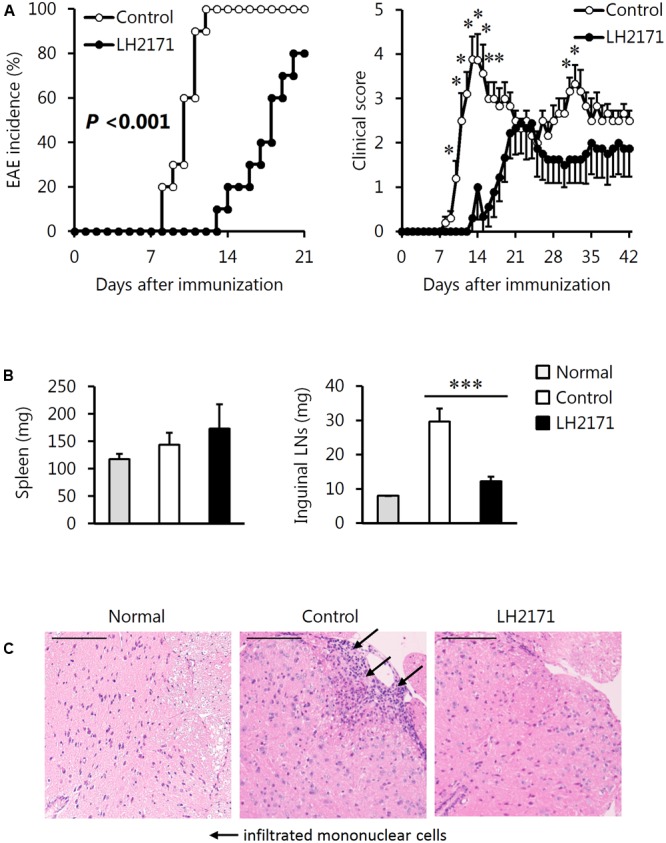
Alleviative effects of LH2171 on the incidence and symptoms of EAE in mice. EAE was induced in SJL/J mice by immunization with a major myelin protein; proteolipid protein. Mice were intraperitoneally injected with either LH2171 or PBS as a vehicle (control). **(A)** After the immunization, mice were observed for measurement of EAE incidence and clinical score. On day 42 after immunization, the spleen, inguinal LNs, draining LNs in this study, and spinal cord were collected from EAE mice; the weights of the spleen and LNs **(B)** were measured. Data are shown as means ± SEM (normal group, *n* = 3; control group, *n* = 10; LH2171-administered group, *n* = 10). The EAE incidence between LH2171-administered mice and control mice was compared with the log-rank test, and the clinical store and tissue weight were compared between groups with Student’s *t*-test (^∗^*P* < 0.05, ^∗∗∗^*P* < 0.001). **(C)** The spinal cord sections obtained from the mice on day 42 after immunization were analyzed by haematoxylin and eosin staining. Scale bar = 100 μm.

We examined the efficacy of LH2171 administration to attenuate EAE in mice. The mice were immunized with an emulsion of proteolipid protein peptide (PLP139–151) and CFA in addition to two intraperitoneal injections of pertussis toxin on days 0 and 2 for EAE induction according to the experimental schedule shown in **Figure 1**. The intraperitoneal administration of LH2171 significantly decreased the incidence of EAE, clinical score, and enlargement of the inguinal LNs (the draining LNs in the model) when compared to the control (vehicle-only treatment) EAE mice (**Figures 2A,B**). Furthermore, histological examination of the spinal cord revealed a relatively lower number of infiltrated mononuclear cells in the mice administered LH2171 than in the control mice (**Figure 2C**).

### LH2171 Reduced the Number of Pathogenic Immune Cells in the Spinal Cord at the Peak Stage of EAE Severity

Next, we evaluated the population and number of Th1 and Th17 cells in the spinal cord of LH2171-administered EAE mice, because these cells have been shown to play several pathogenic roles in the development and progression of EAE (Goverman, 2009).

**FIGURE 3:**
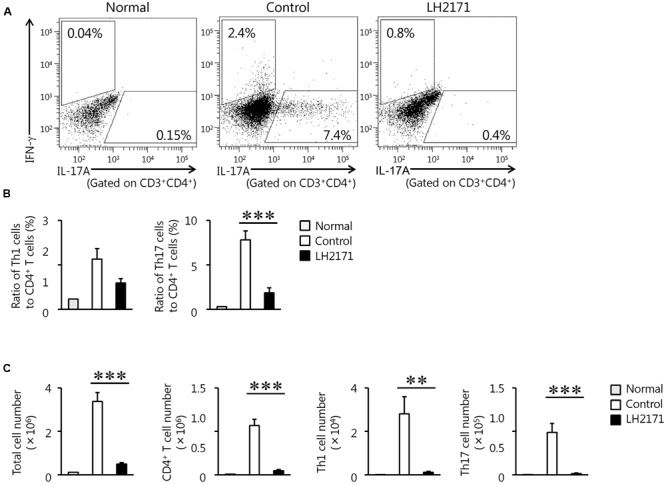
Reduction of the population of pathogenic immune cells in the spinal cord by LH2171 administration at the peak stage of EAE. The spinal cord was collected from normal mice and EAE mice treated with or without LH2171 on day 14, at the peak stage of disease activity. Immune cells isolated from the spinal cord were analyzed by flow cytometry. CD3^+^ CD4^+^ IFN-γ^+^ cells were identified as Th1 cells and gated with an upper-left trapezoid and CD3^+^ CD4^+^ IL-17A^+^ cells were identified as Th17 cells and gated with a lower-right trapezoid **(A)**. The ratio of Th1 and Th17 cells to CD4^+^ T cells **(B)** or the numbers of total lymphocytes, CD4^+^ T cells, Th1 cells, and Th17 cells **(C)** were determined. Data are shown as a means ± SEM (normal group, *n* = 2; control group, *n* = 12; LH2171-treated group, *n* = 11). LH2171-treated mice were compared with control mice by Student’s *t*-test (^∗∗^*P* < 0.01, ^∗∗∗^*P* < 0.001).

Administration of LH2171 significantly decreased the ratio of Th17 cells, but not Th1 cells, to CD4^+^ T cells in the spinal cords obtained from mice at day 14 after the immunization, corresponding to the peak stage of disease activity (**Figures 3A,B**). The numbers of Th17 and Th1 cells in the spinal cord were significantly decreased in the LH2171-administered mice (**Figure 3C**).

### LH2171 Reduced the Number of Th17 Cells in the Inguinal LNs before the Onset of EAE

**FIGURE 4:**
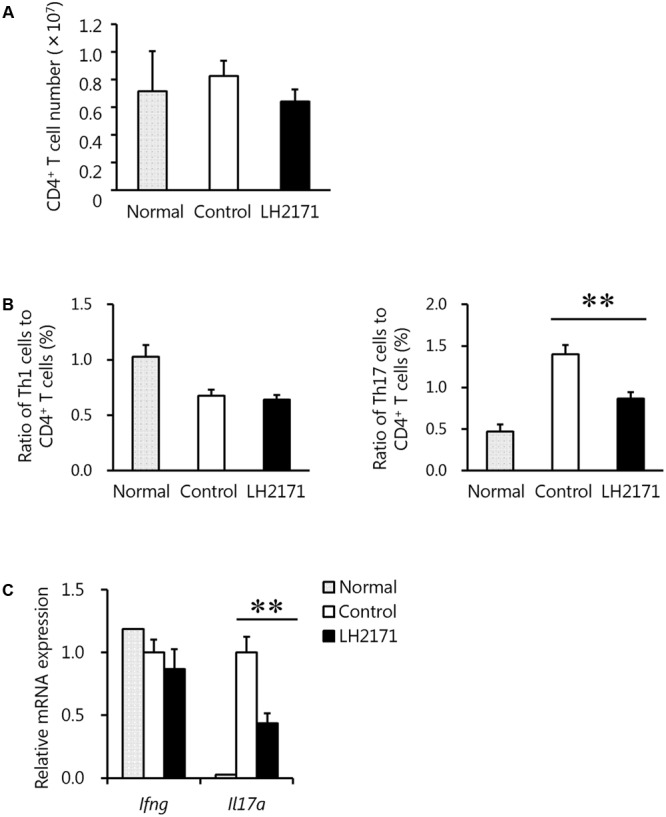
Reduction of the Th17 cell population in the inguinal LNs by LH2171 administration before the onset of EAE. The inguinal LNs were collected from normal mice and EAE mice treated with or without LH2171 on day 7, before EAE onset. Immune cells isolated from the LNs were analyzed by flow cytometry, and the number of CD4^+^ T cells **(A)** and the ratio of Th1 or Th17 cells to CD4^+^ T cells **(B)** were determined. Expression levels of cytokine genes in immune cells from the LNs were determined by qRT-PCR **(C)**. Data are shown as a means ± SEM (normal group, *n* = 2; control group, *n* = 12; LH2171-treated group, *n* = 10). LH2171-treated mice were compared with control mice by Student’s *t*-test (^∗∗^*P* < 0.01).

Pathogenic cells in the LNs of EAE models are activated to invade the surrounding CNS tissues such as the brain and spinal cords (Ito et al., 2014). Therefore, to reveal the mechanism by which LH2171 relieved the incidence and symptoms of EAE while suppressing the infiltration of pathogenic immune cells such as Th17 and Th1 cells into the spinal cord, we further investigated these immune cells in the inguinal LNs. At day 7 after the immunization, when none of the mice had yet developed EAE (**Figure 2A**), the number of CD4^+^ cells in the spinal cords of the LH2171-administered mice showed a tendency to decrease (**Figure 4A**) and the ratio of Th17, but not Th1 cells, to CD4^+^ cells was significantly lower than that of the control mice with a comparable level to that of the normal (not immunized) mice (**Figure 4B**). Furthermore, the expression of cytokine genes showed the same tendency as the ratios of Th17 and Th1 cells to CD4^+^ cells, in which the expression of *Il17a*, but not *Ifng*, was lower in the LNs of LH2171-administered mice (**Figure 4C**). These findings suggested that LH2171 might suppress the differentiation of naïve CD4^+^ cells to Th17 cells in the draining LNs.

### LH2171 Administration Regulated the Expression of Cytokines and Transcription Factors Involved in Th17 Differentiation in the Inguinal LNs before EAE Onset

**FIGURE 5:**
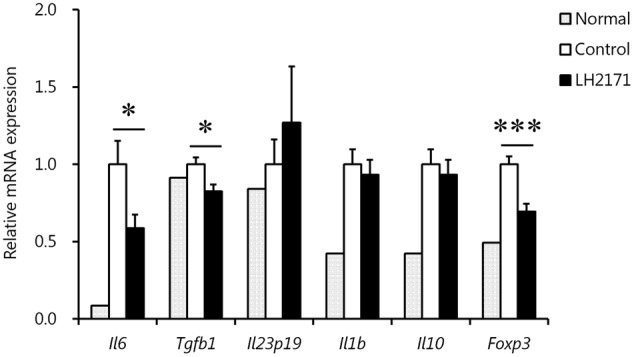
Regulation of the expression of cytokines and transcription factors involved in Th17 differentiation in the inguinal LNs by LH2171 administration before EAE onset. The inguinal LNs were collected from normal mice and EAE mice treated with or without LH2171 on day 7, before EAE onset. Data are shown as means ± SEM (normal group, *n* = 2; control group, *n* = 12; LH2171-treated group, *n* = 10). LH2171-treated mice were compared with control mice by Student’s *t*-test (^∗^*P* < 0.05, ^∗∗∗^*P* < 0.001).

To clarify why LH2171 specifically decreased the number of Th17 cells, we evaluated the expression levels of cytokines and transcription factors related to Th17 differentiation in the LNs before EAE onset. As expected, the expression levels of *Il6* and *Tgfb1*, which promote Th17 differentiation (Leung et al., 2010), were significantly decreased following LH2171 administration (**Figure 5**). In particular, the *Il6* expression level showed obvious reduction, whereas the expression levels of *Il10* and *Foxp3*, which suppress Th17 differentiation (Zhang et al., 2004), did not change or decrease by LH2171 administration. There was also no difference in the expression of chemokine ligand 20 (*Ccl20*) in the spinal cord or chemokine receptor (CCR) 2, CCR4, and CCR6 expression on Th17 cells in the draining LNs of LH2171-administered mice compared to the control mice (Supplementary Figure S1).

### LH2171 Inhibited IL-6 Production from LPS-Stimulated Antigen-Presenting Cell Lines

**FIGURE 6:**
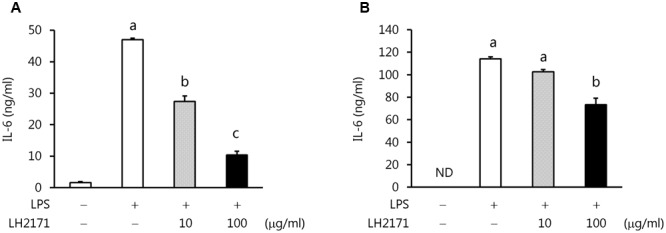
Inhibition of IL-6 production from LPS-stimulated DC2.4 and RAW264.7 cells by LH2171. DC2.4 cells **(A)** or RAW 264.7 cells **(B)** were incubated with or without LH2171 for 4 h, and then stimulated with LPS for 20 h. The amount of IL-6 produced was measured in the culture supernatants by enzyme-linked immunosorbent assay. Data are shown as means ± SEM (*n* = 3). Statistical differences between the groups were analyzed using the Tukey–Kramer test and significant differences (*P* < 0.05) are represented by different letters (a–c).

To more precisely evaluate this clear inhibitory effect of LH2171 on IL-6 production, we examined whether LH2171 could suppress IL-6 production from the major IL-6-producing cells dendritic cells (DC2.4) and macrophages (RAW 264.7). The cells were treated with LH2171 and then stimulated with LPS, and the amounts of IL-6 produced in the cell culture supernatants were measured. Indeed, LH2171 significantly suppressed IL-6 production from both LPS-stimulated DC2.4 and RAW 264.7 cells in a concentration-dependent manner (**Figure 6**).

## Discussion

Multiple sclerosis is an inflammatory demyelinating autoimmune disease of the CNS resulting in various symptoms depending on the lesion areas in the CNS, and significantly erodes the patients’ quality of life (Brownlee et al., 2017; Ontaneda et al., 2017). For example, MS can lead to optic nerve disorders contributing to visual loss, cerebellar deficits leading to a gait deficit and shaking of a limb, disorders of the spinal cord leading to numbness of the abdomen and chest, and cerebral lesions leading to paresthesia of the limbs or movement disorders, as well as effects on cognitive function (Gensicke et al., 2012; Lee and Han, 2015). MS is most commonly treated with oral steroids and immunosuppressive drugs, but their long-term use could carry a risk of serious side effects such as severe infections and the development of certain types of cancer. Furthermore, the effectiveness of these drugs is limited to a short window, only after the onset of MS. Therefore, identification of any foods or supplements that can be safely taken on a daily basis would be of great value for the prevention of MS before its onset.

A few previous studies have demonstrated the preventive effects of LAB on EAE, a murine model of MS (Lavasani et al., 2010; Takata et al., 2011). For example, *Pediococcus acidilactici* (Takata et al., 2011) and a mixture of the three lactobacilli strains (*L. paracasei* DSM 13434, *L. plantarum* DSM 15312, and DSM 15313) reduced inflammation in the CNS of EAE mice (Lavasani et al., 2010), which was accompanied by the enhanced production of IL-10, one of the major anti-inflammatory cytokines. However, in our study, although the administration of LH2171 decreased the incidence and alleviated the symptoms of EAE, it did not influence IL-10 expression, suggesting that the preventive effect of LH2171 might be exerted through a different mechanism.

In this study, LH2171 administration to EAE mice significantly suppressed the infiltration of pathogenic T cells, especially Th17 cells, into the spinal cord. Th17 cells are known to contribute to CNS demyelination via activation of other inflammatory immune cells, and Th17-producing IL-17 impairs the integrity of the blood–brain barrier, permitting entry of circulating immune cells into the CNS while stimulating astrocytes and microglia to produce inflammatory mediators (Ransohoff and Brown, 2012). CCR2, CCR4, CCR6, and CCL20, one of the ligands for CCR6, play important roles in the infiltration of Th17 cells to the CNS from the peripheral tissue (Kohler et al., 2003; Acosta-Rodriguez et al., 2007; Reboldi et al., 2009; Arima et al., 2012). In addition, Th17 cells, which show high cell-surface expression of CCR2, CCR4, and CCR6, are attracted to CCLs localizing in CNS tissues, such as CCL20, and invade the CNS (Kohler et al., 2003). However, there was no effect of LH2171 administration before the onset of EAE on Ccl20 expression in the spinal cord or on CCR2, CCR4, and CCR6 expression on Th17 cells in the draining LNs. These findings suggest that LH2171 does not promote the migration of peripheral Th17 cells into the spinal cord, but might instead suppress the differentiation of Th17 cells to subsequently reduce the cell number in the peripheral tissue, thereby indirectly inhibiting the invasion of Th17 cells into the spinal cord. In support of this mechanism, administration of LH2171 decreased the ratio of Th17 cells to CD4^+^ T cells in the draining LNs before the onset of EAE, which strongly suggested that LH2171 could indeed suppress Th17 cell differentiation. By contrast, although the ratio of Th1 cells to CD4^+^ T cells also has reported pathogenicity for EAE and MS similar to the ratio of Th17 cells to CD4^+^ T cells (Goverman, 2009; Domingues et al., 2010), LH2171 administration did not decrease the Th1 cell to CD4^+^ T cell ratio, thus suggesting that LH2171 might have no influence on Th1 cell differentiation.

A variety of cytokines and immune cells are implicated in Th17 differentiation, leading to the development of EAE and MS (Bhat and Steinman, 2009; Goverman, 2009; Domingues et al., 2010; Segal, 2010). IL-6 in particular is known to play an important role in Th17 differentiation, and is indispensable for turning naïve T cells into Th17 cells (Nishikawa et al., 2008; Leung et al., 2010). Development of Th17 cells is driven by the signaling pathways mediated by TGF-β, IL-6, IL-23, IL-1β, and signal transducer and activator of transcription 3 (STAT3). Binding of IL-6 to its receptor activates STAT3, which then induces the expression of RAR-related orphan receptor gamma (ROR-γt), a specific transcription factor whose expression is restricted to the IL-17-producing Th17 cell subset alone, and STAT3 signaling also restrains the Foxp3-mediated inhibition of ROR-γt (Gaffen et al., 2014; Nalbant and Eskier, 2016). TGF-β induces ROR-γt expression through SMAD phosphorylation and induces IL-23R and IL-1R expression in naïve T cells, rendering them receptive to IL-23 and IL-1β. ROR-γt and STAT3 then bind to the promoters of Th17 cytokines such as IL-17A and IL-17F to induce their transcription. During the later phase, IL-23/IL23R signaling induced by TGF-β and IL-6 further activates STAT3 signaling and ROR-γt expression to stabilize the Th17 cell phenotype.

Therefore, we evaluated the expression of Th17-promoting cytokines such as IL-6, TGF-β, IL-23, and IL-1β, as well as Foxp3 as a Th17-suppressive factor. The results showed that LH2171 suppressed the expression of *Il6* and *Tgfb1*, but not *Il23p19* or *Il1b*, in the draining LNs. Given that the reduction level of *Il6* expression was the most extreme, we carried out further examinations to evaluate whether LH2171 could suppress IL-6 production *in vitro*. Treatment of LH2171 cells to dendritic cells and macrophages, which are major IL-6-producing cells, followed by LPS stimulation to induce IL-6 production showed that LH2171 suppressed IL-6 production from both cell lines in a concentration-dependent manner. In a previous *in vitro* study, we showed that LH2171 could inhibit the production of inflammatory cytokines, including IL-6, in primary murine immune cells (Yamashita et al., 2014). Other studies have also reported the suppressive effects of LAB on IL-6 production *in vitro* and *in vivo* (Griet et al., 2014; Novotny Núñez et al., 2015). Based on these previous findings, LH2171 might suppress the production of IL-6 in EAE mice, leading to inhibition of the differentiation of Th17 cells and their subsequent invasion along with other pathogenic immune cells into the CNS. In this study, we clarified the alleviative effect of intraperitoneal administration of LH2171 on the symptoms of EAE mice. Toward the clinical application, the effect of oral administration of LH2171 on the symptom and gut microbiome of EAE mice remains to be investigated, since it have been reported in recent years that intestinal bacterial flora have a great influence on the symptoms of EAE models (Lavasani et al., 2010; Berer et al., 2011) and MS patients (Miyake et al., 2015).

## Conclusion

Our study demonstrates that the intraperitoneal administration of LH2171 significantly decreased the incidence and attenuated the symptoms of EAE by reducing the number of pathogenic immune cells, including Th17 cells, in the spinal cord. Furthermore, *in vivo* and *in vitro* experiments both showed that LH2171 reduced the expression level and production of Th17-inducing cytokines such as IL-6, which closely correlated with the preventive effects of LH2171 on EAE. These findings suggest that LH2171 might exert a preventive or attenuative effect for patients with autoimmune diseases, including MS.

## Author Contributions

MY, KU, FS, SK, YA, MM, HN, and TM designed the research. MY, KU, TH, FS, and YM performed the experimental work and analyzed the data. MY and TM wrote the manuscript.

## Conflict of Interest Statement

MY, KU, TH, and FS are employees of Megmilk Snow Brand Co., Ltd. The other authors declare that the research was conducted in the absence of any commercial or financial relationships that could be construed as a potential conflict of interest.

## Abbreviations

CCL: chemokine ligand
CCR: chemokine receptor
CFA: complete Freund’s adjuvant
CNS: central nervous systems
EAE: experimental autoimmune encephalomyelitis
IFN: interferon
IL: interleukin
LAB: lactic acid bacteria
LPS: lipopolysaccharide
LH2171: *Lactobacillus helveticus* SBT2171
LNs: lymph nodes
MS: multiple sclerosis
TGF: transforming growth factor
Th: T helper
