# Supplementary material for: Lactobacillus helveticus SBT2171 Attenuates Experimental Autoimmune Encephalomyelitis in Mice
Source: Front Microbiol. 2018 Jan 22;8:2596. doi: 10.3389/fmicb.2017.02596 (PMC5786557; doi:10.3389/fmicb.2017.02596)
Supplement: Supplementary file 1 [file Data_Sheet_1.doc]

Supplementary Material

# *Lactobacillus helveticus* SBT2171 attenuates experimental autoimmune encephalomyelitis in mice

Maya Yamashita, Ken Ukibe, Yumi Matsubara, Tomohiro Hosoya, Fumihiko Sakai, Shigeyuki Kon, Yasunobu Arima, Masaaki Murakami, Hisako Nakagawa, Tadaaki Miyazaki*

*** Correspondence:** Tadaaki Miyazaki: miyazaki@pop.med.hokudai.ac.jp

**Supplementary Methods**

***Antibodies***

The following fluorochrome-labeled anti-mouse antibodies were used for FACS analyses: anti-CD3 (145-2C11), anti-CD4 (GK1.5), anti-IL-17A (TC11-18H10.1), anti-IFN-γ (XMG1.2), anti-CCR4 (2G12), anti-CCR6 (29-2L17) (BioLegend, San Diego, CA), anti-CCR2 (475301) (R&D Systems, Minneapolis, MN).

***Gene expression analysis***

Total RNA was isolated from the inguinal LNs and the spinal cord of EAE mice using the TRIzol reagent (Life technologies, Thermo Scientific, Waltham, MA). RNA was reverse-transcribed into complementary DNA (cDNA) using the ReverTra Ace qPCR kit (TOYOBO, Osaka, Japan). Real-time PCR reactions were performed using the KAPA SYBR FAST Universal qPCR Kit (KAPA BIOSYSTEMS, Boston, MA) according to manufacturer’s instructions. Sequences of the PCR primers are shown in Supplementary Table 1. Data were normalized to the *Actb* gene expression.

***Cell staining and flow cytometric analysis***

To prepare single-cell suspension from inguinal LNs, the LNs of mice were collected and mechanically disrupted in RPMI 1640 culture medium (Wako, Osaka, Japan) containing 10% heat-inactivated fetal bovine serum (GIBCO, Thermo Fisher Scientific, Waltham, MA), 100 U/ml of penicillin, 100 μg/ml of streptomycin (Sigma-Aldrich, St. Louis, MO), and 0.05 mM 2-mercaptoethanol. Cell suspensions were filtered through a 100-μm cell strainer (BD Biosciences), washed twice with the medium, and resuspended in the cell culture medium.

The following markers were used to determine the type of immune cells: CD4^+^ T cells (CD3^+^ CD4^+^), Th17 cells (CD3^+^ CD4^+^ IL-17A^+^). For the Th17 staining, the cells were stimulated with PMA (50 ng/mL) and ionomycin (500 ng/mL) in the presence of brefeldin A (1 µL/mL) (GolgiPlug; BD Biosciences, Heidelberg, Germany) for 5.5 h. Subsequently, the cells were stained intracellularly with anti-IFN-γ and anti-IL-17A antibodies using the BD Cytoﬁx/Cytoperm reagents (BD Biosciences) according to the manufacturer’s protocols. Cytometric data were acquired using a FACSCanto II flow cytometer and analyzed with FACSDiva software (BD Biosciences).

**Supplementary Figure**

**
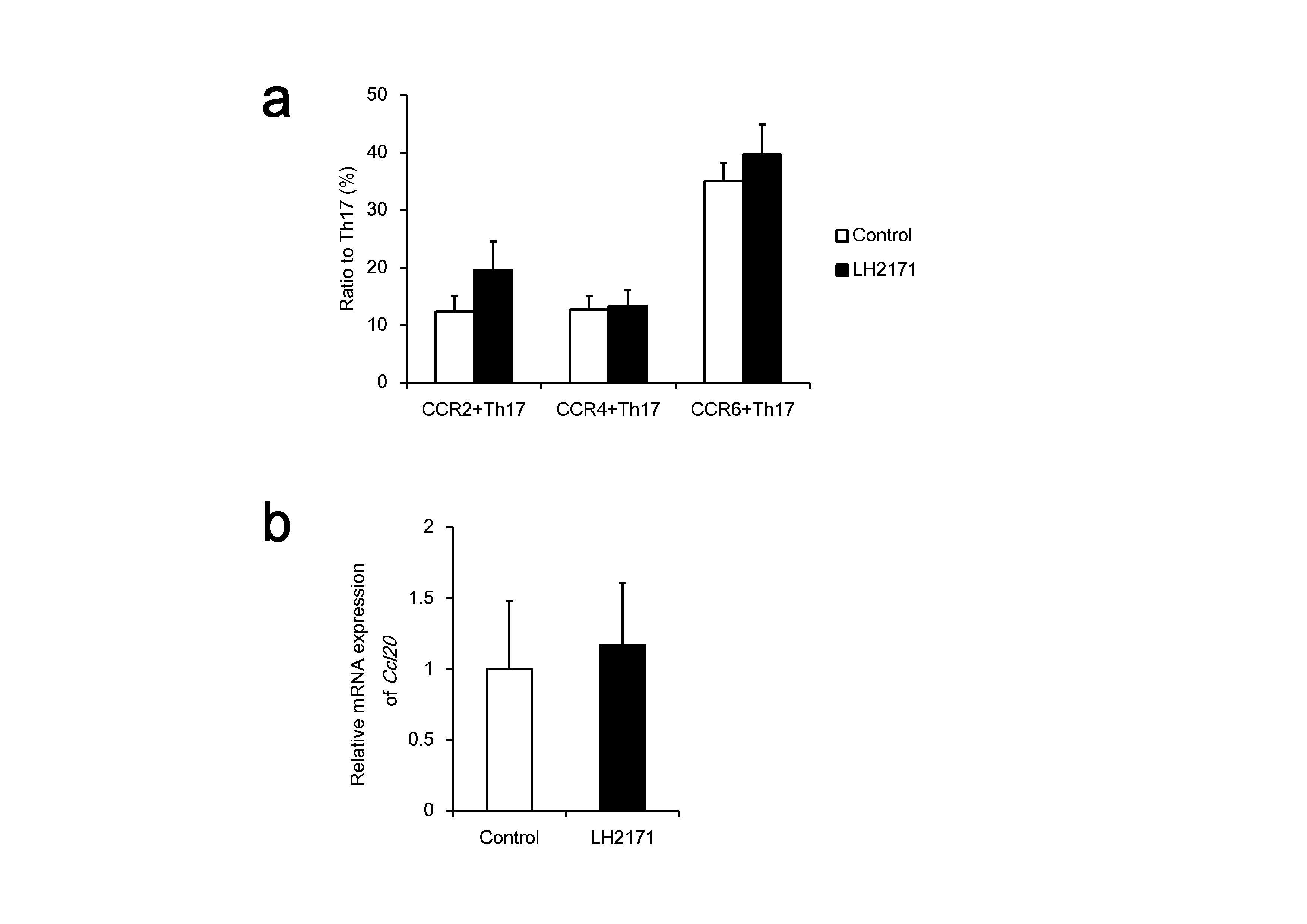
**

**Supplementary Figure S1**

**The ratio of CCR2^+^ Th17, CCR4^+^ Th17, and CCR6^+^ Th17 cells to Th17 cells population in inguinal LNs, and relative expression of *Ccl20* in the spinal cord of LH2171-administrated EAE mice before the onset.**

The inguinal LNs and spinal cord were collected from EAE mice treated with or without LH2171 on day 7, before EAE onset. Immune cells isolated from the LNs were analyzed by flow cytometory, and a ratio of CCR2^+^ Th17, CCR4^+^ Th17, and CCR6^+^ Th17 cells to Th17 cells were determined (a). Expression of *Ccl20* genes in immune cells from the spinal cord were determined by qRT-PCR (b). Data are shown as a means ± SEM (Normal group; n=2, Control group; n=12, LH2171-administrated group; n=10). LH2171-treated mice were compared with control mice by Student's *t*-test.

**Supplementary Table**

**Supplementary Table 1** Primers used in the gene expression analysis.

| Gene Symbol | Forward Primer 5′→3′ | Reverse Primer 5′→3′ |
| --- | --- | --- |
| *Actb* | GGCTGTATTCCCCTCCATCG | CCAGTTGGTAACAATGCCATGT |
| *Ccl20* | CAGAAGCAGCAAGCAACTACGA | CTGTCTTGTGAAACCCACTAGC |
